# Polymeric PEG-based bioorthogonal triggers for prodrug activation in breast cancer†

**DOI:** 10.1039/d4ra08758e

**Published:** 2025-03-05

**Authors:** Madonna M. A. Mitry, Helen M. I. Osborn, Francesca Greco

**Affiliations:** a Reading School of Pharmacy, University of Reading Whiteknights Reading RG6 6AD UK f.greco@reading.ac.uk h.m.i.osborn@reading.ac.uk; b Dept. of Pharmaceutical Chemistry, Faculty of Pharmacy, Ain Shams University Cairo 11566 Egypt

## Abstract

Non-toxic prodrugs have proved of great value in medicinal chemistry programmes for cancer, due to their ability to selectively deliver toxic components at tumour sites once they are activated by a localised mechanism. Since activation of the prodrug to afford the toxic drug is a prerequisite for success of the approach, much interest has focused on the localised chemical and enzymatic mechanisms for activating the prodrugs. Bioorthogonal chemistry has positively impacted this area by providing biocompatible reactions that enable on-demand prodrug activation and active drug release. However, to be effective, it is essential that one of the components of the bioorthogonal reaction is localised at the tumour, in order to initiate the on-demand and on-target activation of the prodrug. Polymers such as poly(ethylene glycol) (PEG) are known to target solid tumours by passive targeting *via* the enhanced permeability and retention (EPR) effect. In this paper, the feasibility of derivatising long PEG chains to afford bioorthogonal activators (PEG-azide and PEG-tetrazine) for prodrug activation *via* the Staudinger ligation and the tetrazine ligation reactions, respectively, is evaluated. The molecular weight of the PEG in the activator and the type of linkage in the prodrug moiety were shown to significantly affect the rate of prodrug activation and hence the rate of drug release. *In vitro* cytotoxicity studies on breast cancer cells (MCF-7 and MDA-MB-231) showed ∼68–76% restoration of the parent drug's cytotoxicity for the Staudinger ligation-based prodrug activation strategy, and 100% restoration of the parent drug's cytotoxicity for the tetrazine ligation-based prodrug activation strategy. Restoration of doxorubicin's ability to intercalate with DNA upon activation of the prodrug by the PEG-activators was also demonstrated *via* fluorescence spectroscopy. Moreover, conjugation of the tetrazine bioorthogonal activator to a 10 kDa PEG polymer improved its serum stability in comparison with other reported tetrazine activators that completely lose their stability in serum over the same period of time. The feasibility of the combined passive targeting/bioorthogonal prodrug activation approach has therefore been demonstrated using a range of prodrugs, activation mechanisms, and *in vitro* assays.

## Abstract 2

A new two-component selective breast cancer-targeting prodrug system is introduced based on the Staudinger ligation and tetrazine ligation bioorthogonal reactions and passive targeting.

## Graphical abstract

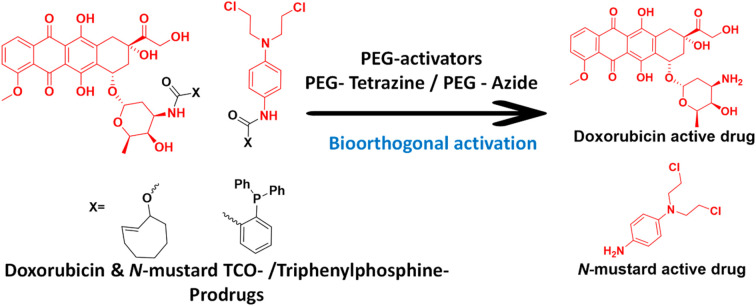

## 1. Introduction

Selective targeting of therapeutic agents to tumour cells is a powerful strategy to increase the effectiveness of cancer therapy. One approach for achieving this is the on-site activation of non-toxic prodrugs through enzymatic catalysis. In theory, this approach can minimize the systemic adverse effects of the chemotherapies by reducing exposure of healthy cells to the toxic drugs. However, off-site hydrolysis and non-specific enzymatic activation can limit the selectivity of this approach.^1–3^ Attention has therefore focused on developing selective prodrug activation approaches that combine *in situ* prodrug activation with targeting mechanisms that deliver the prodrug, or a component that can activate the prodrug, specifically to the tumour.

Bioorthogonal chemistry has emerged as a promising platform for on-demand prodrug activation, as it comprises chemical reactions that can proceed under physiological conditions without interfering with biological processes.^4,5^ The selectivity, specificity, and considerably fast kinetics of these reactions allow precise control of the activation of the non-toxic prodrugs.^6–8^ Many bioorthogonal reactions have been reported to have high potential for selective prodrug activation such as the Staudinger ligation between an azide and a triphenylphosphine,^9^ and the tetrazine ligation between a *trans*-cyclooctene (TCO) and a tetrazine (Tz).^10^ The Staudinger ligation is mainly used for ligation applications due its relatively slow kinetics (*k*_2_ ∼ 10^−3^ M^−1^ S^−1^), and a small number of reports have demonstrated its potential for prodrug activation.^11–13^ The tetrazine ligation is well known for its fast click-to-release reaction kinetics (*k*_2_ ∼ 10^4^ M^−1^ S^−1^) at low concentrations and many reports have demonstrated that the reactivity of the Tz moiety, and consequently the reaction kinetics, can be fine-tuned by altering the properties *e.g.* size/electron density of the Tz moiety.^14–16^

Complementary targeting approaches are engaged to selectively deliver the prodrug or the activator to tumours to maximise the impact of the on target prodrug activation.^7^ These targeting approaches include metabolic glycoengineering (MGE),^11^ active targeting,^17–19^ and passive targeting.^20^ Passive targeting is a promising approach to target solid tumour as it relies on the EPR effect (the enhanced permeability and retention of nanosystems in tumours).^21,22^ This mechanism allows the passive delivery and accumulation of relatively large molecules like polymers, liposomes, and proteins hence rendering the EPR effect a valuable cancer-targeting mechanism.^22–24^

**Fig. 1:**
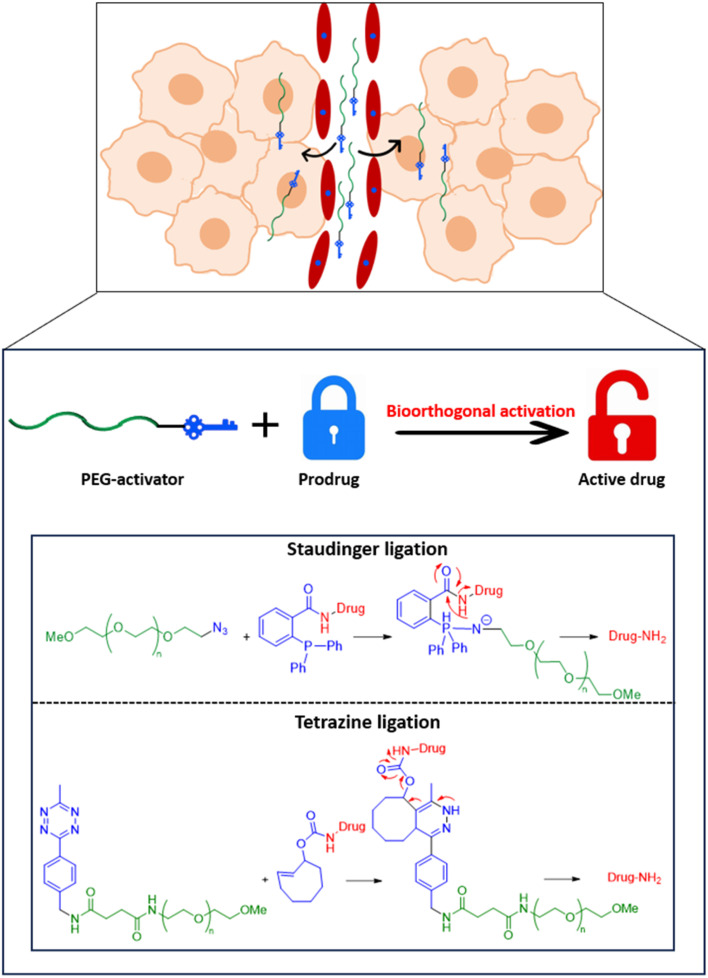
Schematic illustration of prodrug activation allowing release of the toxic drug specifically at the tumour cell by (i) delivery of the PEG-activator component to solid tumours through the EPR effect, (ii) activation of the prodrugs, that are purposefully designed to mask the toxicity of the active drug, using the PEG-activator through bioorthogonal Staudinger ligation and tetrazine ligation reactions.

In this paper, we propose the derivatization of poly(ethylene glycol) (PEG) of suitable molecular weight to afford activators that can be incorporated within a passive targeting bioorthogonal prodrug activation strategy for breast cancer underpinned by the EPR effect (Fig. 1). If successful, this would allow the prodrugs (that are purposefully designed to mask the toxicity of the active drug) to be activated at the required site to afford the active drug *in situ*, hence addressing the limitations of traditional chemotherapy approaches. PEG was selected as the polymeric component due its documented aqueous solubility, high safety profile (almost inert reactivity with blood components), low antigenic and immunogenic properties, good pharmacokinetics, and approval by the FDA for use in humans.^25–28^ Short PEG chains (<10 kDa) have already proved effective in bioorthogonal applications where they were utilised in coating carbon nanotubes and gold nanoparticles for bioorthogonal synthetic, diagnostic or therapeutic purposes.^29–33^ Longer PEG chains (≥10 kDa) are reported to be suitable for use in cancer targeting by the EPR effect due to their good accumulation levels in solid tumours.^34–37^ Therefore, herein, the feasibility of using PEG derivatives of larger molecular weights (≥10 kDa) in bioorthogonal Staudinger ligation- and tetrazine ligation-based prodrug activation is probed. PEG polymers derivatised with azide or Tz moieties were firstly tested for their feasibility to activate complementary prodrugs *via* the Staudinger ligation or tetrazine ligation bioorthogonal reactions, respectively, through HPLC-monitored release studies. Then, prodrug activation and restoration of the antiproliferative activity of the active drugs, as required for localised cancer therapy, was confirmed *in vitro* using MCF-7 and MDA-MB-231 breast cancer cells.

## 2. Results and discussion

### 2.1. Chemical synthesis and proof-of-concept prodrug activation studies demonstrating release of the drug

#### 2.1.1 PEG-activators

To test the feasibility of using the polymer triggers to activate prodrugs to effect drug release by the Staudinger ligation and tetrazine ligation reactions, two polymeric activators were employed, specifically a PEG-azide and a PEG-Tz, respectively. It is critical to take into consideration the molecular weight of the PEG used according to the purpose. PEGs with low molecular weights (<400 Da) are reported to produce toxic hydroxy acids and diacid metabolites due to their ease of metabolism *in vivo*,^26,38^ while PEGs with high molecular weights (≥40 kDa) tend to accumulate in the liver.^25^ Therefore, PEGs with MW between 10–40 kDa are considered more suitable to use for selective tumour delivery by the EPR effect.^34–37^

**Scheme 1:**
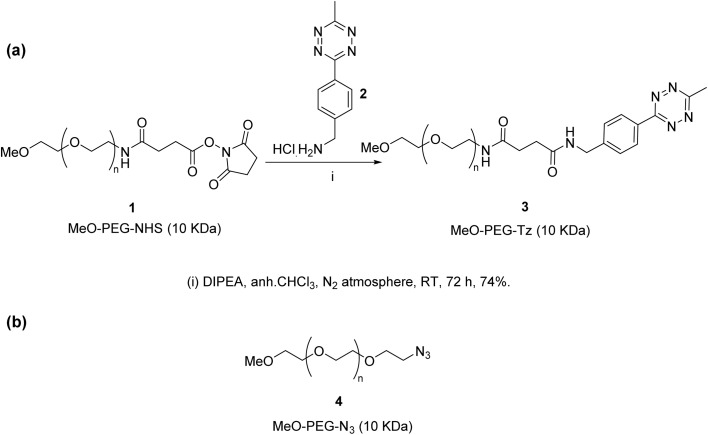
(a) Synthesis of PEG-Tz activator 3. Tz loading: 1.78% W/W, free Tz < 0.2% of total Tz. (b) Chemical structure of PEG-azide activator 4.

To design the PEG-Tz activator, the structure of the employed Tz moiety to be conjugated to the PEG was also considered. Electron withdrawing substituents on the Tz ring are shown to increase the reaction rate but decrease the serum stability of the Tz moiety, while electron donating substituents have the opposite effects.^15^ Therefore, a Tz ring substituted with a 3-benzyl group and a 6-methyl group is proposed to have a good balance between the reaction kinetics and the serum stability and hence was selected for conjugation to the PEG. The synthesis of the PEG-Tz activator 3 was carried out as shown in Scheme 1a ^39^ using commercially available MeO-PEG-NHS (10 kDa) to afford the conjugate in 74%. The conjugate was purified by dialysis and characterised using ^1^H and ^13^C NMR spectroscopy. The average Tz content in the conjugate, as determined *via*^1^H-NMR spectroscopic analysis, was 1.78% w/w, and the amount of free Tz was determined to be <0.2% using RP-HPLC. The commercially available methoxy-azido-PEG was used as the PEG-azide activator, the structure for this is shown in Scheme 1b.

#### 2.1.2 Synthesis of the triphenylphosphine model ester prodrug and doxorubicin and *N*-mustard amide prodrugs

**Scheme 2:**
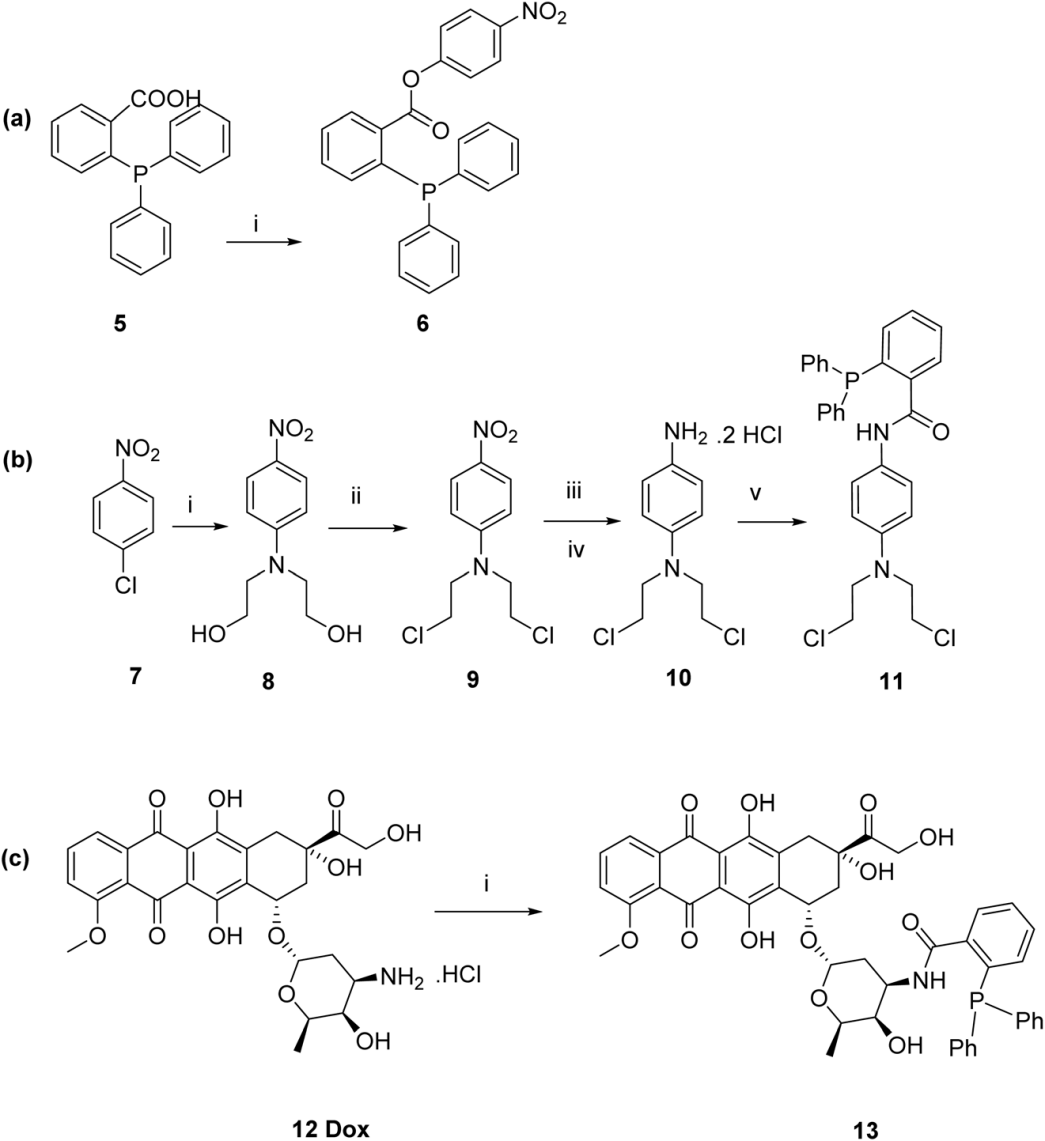
(a) Synthesis of triphenylphosphine model ester prodrug 6. (b) Synthesis of triphenylphosphine-*N*-mustard prodrug 11. (c) Synthesis of triphenylphosphine-doxorubicin prodrug 13.

A model prodrug was initially used where a 4-nitrophenol moiety was used as a substitute for the cytotoxic drug moiety. 4-Nitrophenol was linked by an ester bond to the triphenylphosphine to yield a triphenylphosphine model ester prodrug 6 for the Staudinger ligation prodrug activation approach. The synthesis was carried out as shown in Scheme 2a ^9^ through DCC coupling-mediated Steglich esterification of diphenylphosphanyl benzoic acid 5 with 4-nitrophenol to yield the model ester prodrug 6 in 38%. The compound was purified by column chromatography and characterised using ^1^H and ^13^C NMR spectroscopy, and mass spectrometry. For prodrugs containing cytotoxic components, doxorubicin and nitrogen mustard were selected to be derivatised as bioorthogonal cleavable prodrugs due to their cytotoxicity against breast cancer cells.^40–42^ The prodrugs were designed to comprise the cytotoxic drug attached to the triphenylphosphine masking moiety in a way that would render the prodrugs non-toxic, as required. Triphenylphosphine-*N*-mustard prodrug 11 and triphenylphosphine-doxorubicin prodrug 13 were synthesized according to our reported procedures as shown in Schemes 2b and c.^11^

A RP-HPLC-monitored release study was then conducted to test the feasibility of using PEG-azide to activate the triphenylphosphine-prodrugs to effect drug release in a selective manner. Release of the active drugs from the prodrugs under these specific conditions is essential in order for the prodrugs to afford the cytotoxic drugs at the tumour in a selective manner.

#### 2.1.3 Activation of triphenylphosphine model ester prodrug to release 4-nitrophenol by PEG-azide activators *via* the Staudinger ligation

**Fig. 2:**
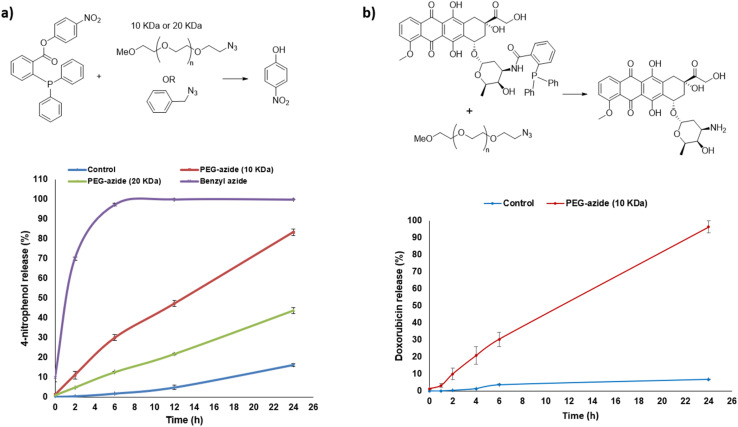
(a) Effect of polymer molecular weight on release rate of 4-nitrophenol from model triphenylphosphine-prodrug 6*via* the Staudinger ligation reaction, release profile was monitored by HPLC as a function of time upon incubation of 6 with activators PEG-azide (10 kDa) or PEG-azide (20 kDa) or benzyl azide or without activators (control). (b) Release profile summary of doxorubicin 12 from triphenylphosphine-Dox prodrug 13*via* the Staudinger ligation reaction monitored by HPLC as a function of time upon its incubation with activator PEG-azide (10 kDa) 4 or without 4 (control). Data are presented as mean ± SEM (*n* = 3).

Two commercially available PEG-azides (10 and 20 kDa) were tested alongside a small molecule azide activator (*i.e.* benzyl azide) as a comparator to ascertain whether the sterics of the polymer would negatively impact the activation reaction. The release of 4-nitrophenol from the triphenylphosphine model ester prodrug 6 using three different azide activators was monitored by HPLC over 24 hours in aqueous condition (H_2_O : CH_3_CN, 1 : 1 v/v) at 37 °C. After 24 hours, 4-nitrophenol was completely released (100%) upon reaction with the small molecule benzyl azide activator, 83% of 4-nitrophenol was released upon reaction with PEG-azide (10 kDa) and 44% of 4-nitrophenol was released upon reaction with PEG-azide (20 kDa) (Fig. 2a). These results clearly indicate that the size of the activator affects the rate of release and are suggestive that different molecular weights can be selected and further developed depending on the desired application and kinetics of release (*i.e.* using a PEG of higher molecular weight for slow or sustained release). As the 10 kDa size showed faster activation rate, and is considered as a suitable molecular weight for use within drug release strategies based on the EPR effect, it was selected for subsequent prodrug activation and release studies and *in vitro* biological studies.

#### 2.1.4 Activation of triphenylphosphine-doxorubicin prodrug to release doxorubicin by PEG-azide activator *via* the Staudinger ligation

To test the feasibility of the PEG-azide for activation of the prodrugs that are purposefully designed to mask the toxicity of the free drug, to afford the cytotoxic component, the triphenylphosphine-doxorubicin prodrug 13 was incubated with the selected PEG-azide 4 activator (10 kDa) and the release of doxorubicin (the active cytotoxic drug) was monitored. The triphenylphosphine-doxorubicin prodrug 13 was selected for the release study as the *N*-mustard moiety that would be released from the triphenylphosphine-*N*-mustard prodrug 11 is unstable making monitoring of the release of the cytotoxic drug difficult. Using the same conditions as trialled previously (H_2_O : CH_3_CN, 1 : 1 v/v) at 37 °C, doxorubicin was completely released (100%) after 24 hours illustrating the effectiveness of the strategy to release the active drug from the prodrug (Fig. 2b).

#### 2.1.5 TCO model carbonate prodrug and doxorubicin and *N*-mustard carbamate prodrugs

**Scheme 3:**
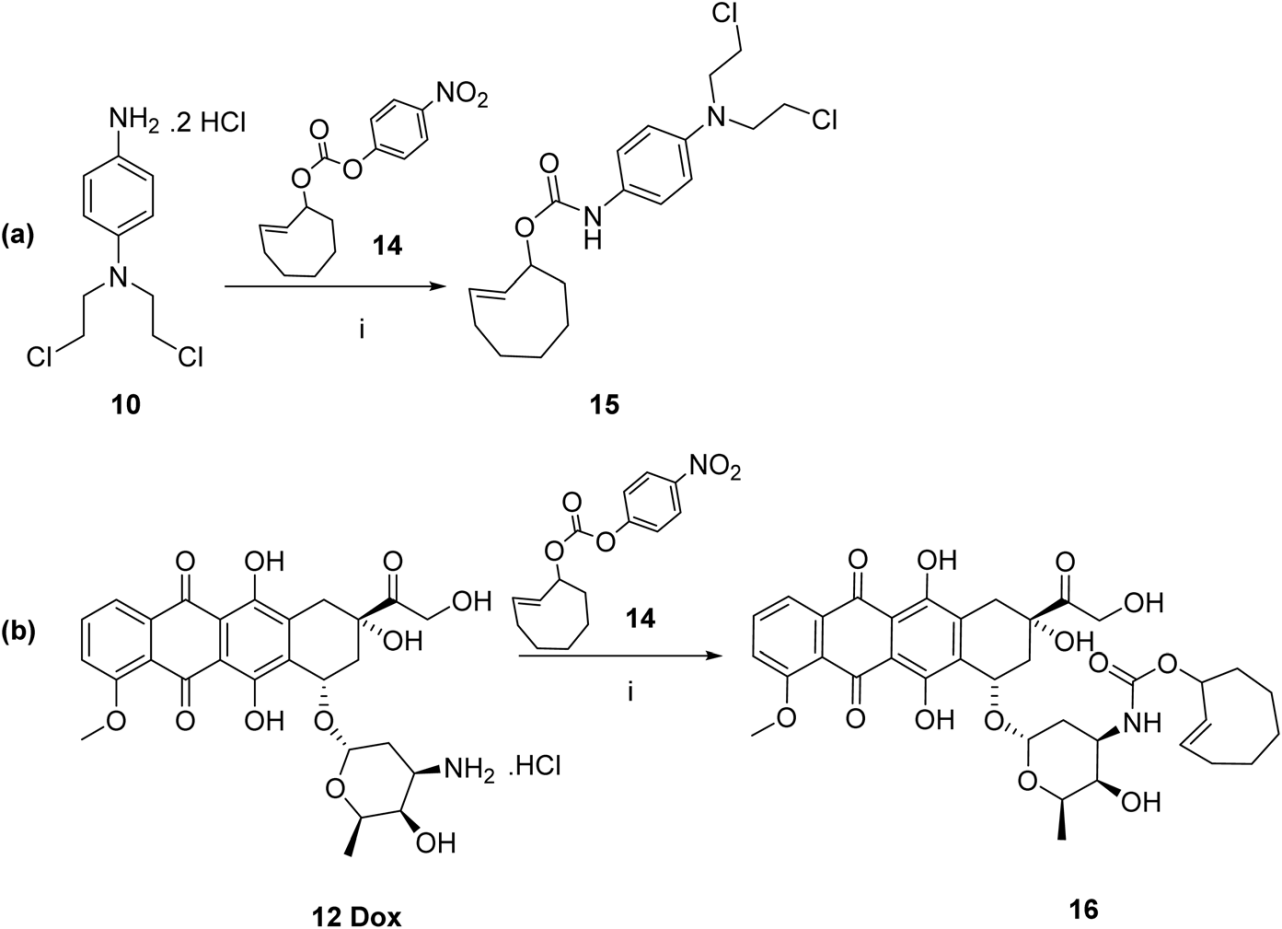
Synthesis of (a) TCO-*N*-mustard prodrug 15 and (b) TCO-*N*-doxorubicin prodrug 16.

A commercially available TCO compound 14 was used as a model prodrug where the 4-nitrophenol was linked by a carbonate bond to the TCO (structure shown in Scheme 3). A novel TCO-*N*-mustard prodrug 15 along with the previously reported TCO-doxorubicin prodrug 16 ^10^ were also synthesized according to our reported procedures as shown in Schemes 3a and b.^43^

Then, to test the feasibility of the PEG-Tz to activate the TCO-prodrugs, a RP-HPLC-monitored release study was conducted.

#### 2.1.6 Activation of TCO-model carbonate prodrug to release 4-nitrophenol by PEG-Tz activator *via* the tetrazine ligation

**Fig. 3:**
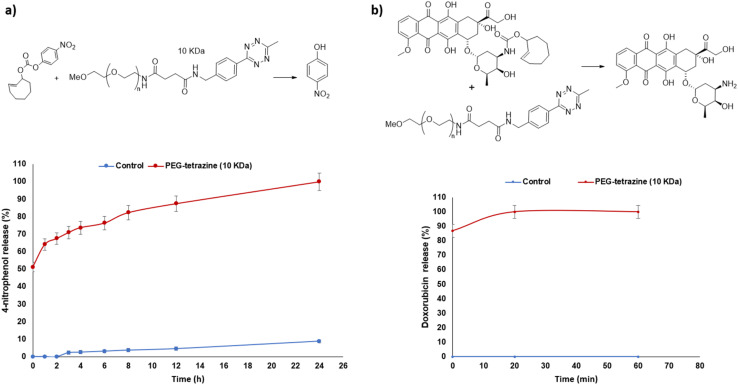
(a) Release profile summary of 4-nitrophenol from model carbonate TCO-prodrug 14*via* the tetrazine ligation reaction monitored by HPLC as a function of time upon its incubation with activator PEG-Tz (10 kDa) 3 or without 3 (control). (b) Release profile summary of doxorubicin 12 from TCO-Dox prodrug 16*via* the tetrazine ligation reaction monitored by HPLC as a function of time upon its incubation with activator PEG-Tz (10 kDa) 3 or without 3 (control). Data are presented as mean ± SEM (*n* = 3).

The release of 4-nitrophenol from the 4-nitrophenyl carbonate TCO model prodrug 14 was monitored by HPLC after its incubation with PEG-Tz (10 kDa) activator 3 under the same previously used aqueous conditions (H_2_O : CH_3_CN, 1 : 1 v/v) at 37 °C. After 24 hours, 4-nitrophenol was completely released (100%) (Fig. 3a).

#### 2.1.7 Activation of TCO-doxorubicin prodrug to release doxorubicin by PEG-Tz activator *via* tetrazine ligation

To test the feasibility of the PEG-Tz for activation of the prodrugs to release the cytotoxic component, the TCO-doxorubicin prodrug 16 was incubated with PEG-Tz 3 activator (10 kDa) and the release of doxorubicin (the active cytotoxic drug) was monitored using the same previously used aqueous conditions (H_2_O : CH_3_CN, 1 : 1 v/v) at 37 °C. Doxorubicin was completely released (100%) after 20 minutes of incubation again illustrating the effectiveness of the strategy to release the active drug from the prodrug (Fig. 3b).

Taken together, these HPLC release studies demonstrate the feasibility of using the 10 kDa Mw PEG-based activators in conjunction with bioorthogonal activation mechanisms to release cytotoxic drugs from triphenylphosphine- or TCO-prodrugs. Studies were therefore progressed to *in vitro* testing in relevant cell models.

### 2.2. Prodrug activation to effect drug release *in vitro*

When selecting an appropriate tumour type in which to test our strategy further, it was important to select one that is documented as being suitable for passive targeting through the EPR effect. Pancreatic, colorectal and breast cancers have been reported to display the highest accumulation ratio of nanosystems in the tumour *versus* normal tissues^44^ and since doxorubicin and *N*-mustards are effective on breast cancer,^45,46^ MCF-7 and MDA-MB-231 breast cancer cell lines were selected to determine the effectiveness of prodrug activation, to afford the cytotoxic drugs, by the PEG derivatives *in vitro*.

The IC_50_ of the triphenylphosphine-Dox prodrug 13 was found to be more than 20-fold higher (4.6 μM and 9.5 μM) than that of the active Dox 12 (0.2 μM and 0.4 μM) in MCF-7 and MDA-MB-231 breast cancer cells, respectively. The IC_50_ of the TCO-Dox prodrug 16 was found to be around 10-fold higher (2.4 μM and 3.5 μM) than that of the active Dox 12 (0.2 μM and 0.4 μM) in MCF-7 and MDA-MB-231 breast cancer cells, respectively (Table 1). Since a higher IC_50_ value correlates with a lower cytotoxicity, this demonstrates that the triphenylphosphine and the TCO moieties are effective for masking the cytotoxic activity of doxorubicin within the prodrug, as required. Moreover, the increased IC_50_ was also indirectly suggestive of prodrug stability in the cell system. For the *N*-mustard based prodrugs, only the IC_50_ of the prodrugs were assessed due to the instability of the active *N*-mustard drug (bis-chloro amino derivative).

**Table 1:** IC_50_ values (μM) for prodrugs 11, 13, 15 and 16 with and without activation in MCF-7 and MDA-MB-231 cells using the MTT assay. Data indicate mean ± SEM (*n* = 3). In the absence of activation, IC_50_ values for prodrugs are higher than for the free drug demonstrating the lower toxicities of the prodrugs. After activation, toxicity is restored as a result of the release of the cytotoxic component from the prodrug

Compounds	IC_50_ (μM)	
	MCF-7	MDA-MB-231
PEG-N_3_ (10 kDa) 4	>150	>150
Doxorubicin 12	0.2 ± 0.03	0.4 ± 0.009
Triphenylphosphine-Dox prodrug 13	4.6 ± 0.19	9.5 ± 0.12
Triphenylphosphine-Dox prodrug 13 + PEG-N_3_4	1.6 ± 0.48	2.6 ± 0.03
Triphenylphosphine-*N*-mustard prodrug 11	20.8 ± 1.27	19.7 ± 0.78
Triphenylphosphine-*N*-mustard prodrug 11 + PEG-N_3_4	4.7 ± 0.32	4.3 ± 0.18
PEG-Tz (10 kDa) 3	>150	>150
Doxorubicin 12	0.2 ± 0.03	0.4 ± 0.009
TCO-Dox prodrug 16	2.4 ± 0.03	3.5 ± 0.44
TCO-Dox prodrug 16 + PEG-Tz 3	0.2 ± 0.02	0.43 ± 0.11
TCO-*N*-mustard prodrug 15	23.1 ± 0.65	30.7 ± 0.96
TCO-*N*-mustard prodrug 15 + PEG-Tz 3	0.7 ± 1.02	2.4 ± 0.26

Next, the cytotoxicities of the prodrugs were assessed in the presence of the polymeric activator. Effective release would result in (at least partial) restoration of the cytotoxicity of doxorubicin. The strategy proved successful to a different extent depending on the prodrug–trigger pair.

**Fig. 4:**
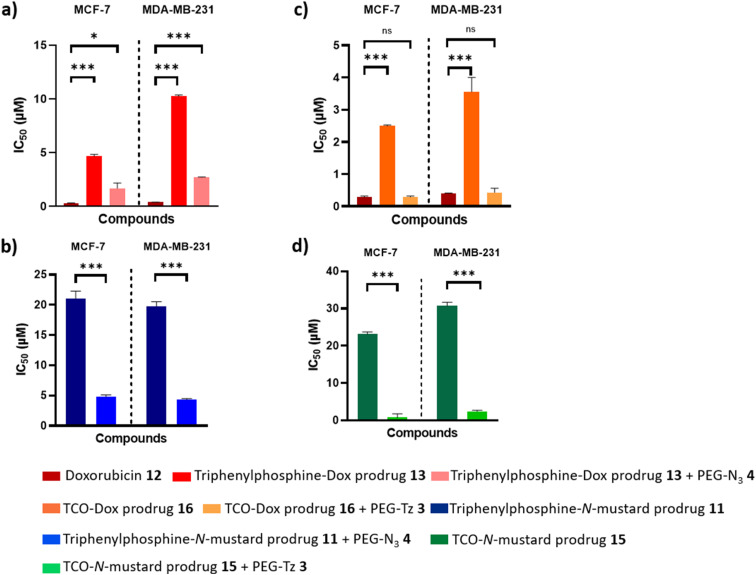
Statistical significance of the prodrugs IC_50_ with or without activator. (a) Determined IC_50_ of Doxorubicin 12 and its triphenylphosphine-prodrug 13 against MCF-7 and MDA-MB-231 cells and IC_50_ of the prodrug 13 after activation by PEG-azide 4. (b) Determined IC_50_ of triphenylphosphine-*N*-mustard prodrug 11 and the prodrug 11 after activation by PEG-azide 4 against MCF-7 and MDA-MB-231 cells. (c) Determined IC_50_ of Doxorubicin 12 and its TCO-prodrug 16 against MCF-7 and MDA-MB-231 cells and IC_50_ of the prodrug 16 after activation by PEG-Tz 3. (d) Determined IC_50_ of TCO-*N*-mustard prodrug 15 and the prodrug 15 after activation by PEG-Tz 3 against MCF-7 and MDA-MB-231 cells. Data are presented as mean ± SEM (*n* = 3). ns represents no significance (*p* > 0.05), * indicates difference at the *p* < 0.05, *** indicates difference at the *p* < 0.001 significance level.

Specifically, pre-treatment of MCF-7 and MDA-MB-231 breast cancer cells with the PEG-Tz activator 3, followed by exposure to the TCO-Dox prodrug 16 resulted in a complete restoration of doxorubicin's cytotoxicity (IC_50_ values 0.2 μM in MCF-7 and 0.4 μM for MDA-MB-231, completely equivalent, *p* > 0.05 to those observed for doxorubicin alone, Table 1 and Fig. 4, S1c and S2c†)*.* With the TCO-*N*-mustard prodrug 15, the IC_50_ was found to be 23.1 μM and 30.7 μM in MCF-7 and MDA-MB-231 breast cancer cells, respectively and after the pre-treatment with the PEG-Tz activator 3, the IC_50_ values decreased to 0.7 μM and 2.4 μM, respectively (***p < 0.001) indicating the successful prodrug activation by the tetrazine ligation reaction (Table 1 and Fig. 4, S1d and S2d†).

Restoration of the cytotoxic activity was also observed for the systems relying on activation by the Staudinger ligation reaction. However, in this case, restoration of activity was only partial. Namely, MCF-7 and MDA-MB-231 breast cancer cells were first pre-treated with the PEG-azide activator 4, and then with the triphenylphosphine-*N*-mustard prodrug 11 or the triphenylphosphine-Dox prodrug 13. The IC_50_ values (Table 1 and Fig. 4, S1a and S2a†) indicated prodrug activation with good levels of restoration of the active doxorubicin's activity, but not complete (IC_50_ 1.6 μM and 2.6 μM *versus* 0.2 μM and 0.4 μM for free doxorubicin) in MCF-7 (*p < 0.05) and MDA-MB-231 (***p < 0.001) breast cancer cells, respectively. The IC_50_ for the triphenylphosphine-*N*-mustard prodrug 11 was found to be 20.8 μM and 19.7 μM in MCF-7 and MDA-MB-231 breast cancer cells, respectively, and after testing the prodrug activation as previously described with the doxorubicin prodrug, these IC_50_ values decreased to 4.7 μM and 4.3 μM, respectively (***p < 0.001) indicating the successful prodrug activation by the Staudinger ligation reaction (Table 1 and Fig. 4, S1b and S2b†).

To further test the safety of the prodrugs 11, 13, 15 and 16 on non-cancerous cells, the IC_50_ of the triphenylphosphine-Dox prodrug 13 and TCO-Dox prodrug 16 were determined on L929 fibroblast cells and were found to be 11.3 μM and 6.4 μM, respectively (2 folds higher than their IC_50_ on MCF-7 cells). The IC_50_ for the triphenylphosphine-*N*-mustard prodrug 11 and the TCO-*N*-mustard prodrug 15 on L929 cells were found to be 31.4 μM and 38 μM, respectively (∼2 folds higher than their IC_50_ on MCF-7 cells). Moreover, the IC_50_ of the PEG-azide 4 and the PEG-Tz 3 on L929 cells were >150 μM. These results therefore further demonstrate the feasibility of using the PEG activation strategy as a selective drug delivery strategy for breast cancer (Fig. S3†).

In summary, the TCO-Tz activation system can be considered to be more advantageous for effecting release of the drug from the prodrug than the azide-triphenylphosphine system, in terms of rate of activation and restoration of active drug cytotoxicity. However, the lower toxicity of the triphenylphosphine-prodrugs, as demonstrated by their higher IC_50_ values on both of the breast cancer cell lines compared with the TCO-prodrugs, would allow higher concentrations to be used. This could potentially further enhance the Staudinger ligation activation rate in future research.

### 2.3. Fluorescence spectroscopy of drug-DNA complexes

**Fig. 5:**
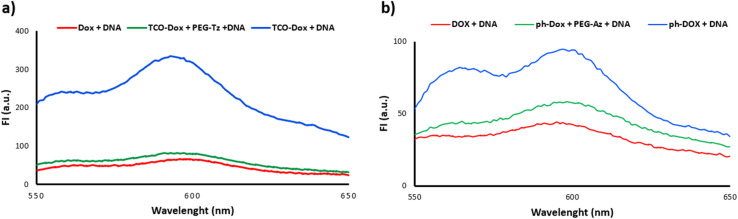
Fluorescence spectra of DNA solution incubated with (a) Dox 12, TCO-Dox 16 and TCO-Dox 16 + PEG-Tz 3. (b) Dox 12, triphenylphosphine-Dox 13 and triphenylphosphine-Dox 13 + PEG-N_3_4.

To confirm the activation of the doxorubicin-prodrugs 13 and 16 by the PEG activators, the DNA intercalation abilities of the prodrugs, and the active doxorubicin, were also tested.^47^ Doxorubicin is an anthracycline drug that intercalates with DNA and suppresses the action of the topoisomerase II enzyme and thereby inhibits the cancer cell replication.^48,49^ Therefore if doxorubicin's mechanism of action is diminished upon forming the prodrug, intercalation with DNA may be reduced. An aqueous solution of DNA (calf thymus) (1 mg mL^−1^) was added to separate samples of doxorubicin 12, TCO-Dox prodrug 16 alone, and TCO-Dox prodrug 16 in the presence of the PEG-Tz 3. In a parallel experiment, the DNA aqueous solution was added to separate samples of doxorubicin 12, triphenylphosphine-Dox prodrug 13 alone, and triphenylphosphine-Dox prodrug 13 which was previously incubated with PEG-azide 4 for 24 hours at 37 °C. The fluorescence of both prodrugs decreased to a comparable level to that of the free doxorubicin, hence demonstrating restoration of the doxorubicin's DNA intercalation activity upon activation of the prodrug by the PEG-activators (Fig. 5).

### 2.4. Serum stability of TCO-*N*-mustard prodrug (15), TCO-doxorubicin prodrug (16) and PEG-Tz activator (3)

In order to determine the suitability of the prodrug/activator system for subsequent use *in vivo*, the stabilities of the TCO prodrugs (*i.e.* TCO-*N*-mustard 15 and TCO-doxorubicin 16) and the complementary PEG-Tz activator 3 in serum were considered. The stabilities of the TCO prodrugs (*i.e.* TCO-*N*-mustard 15 and TCO-doxorubicin 16) were previously reported by ourselves,^43^ with the results demonstrating that the TCO-*N*-mustard prodrug 15 showed very good serum stability with 73 ± 2.5% remaining intact after 12 h of serum incubation. Although the TCO-DOX prodrug 16 was stable for a shorter time (67 ± 0.7% was intact after 2 h of serum incubation but less than 5% at 6 h) this was still considered appropriate for the chosen application due to the complete release of the active drug from this prodrug within 20 minutes, as demonstrated by HPLC release studies herein (Fig. 3b).

**Fig. 6:**
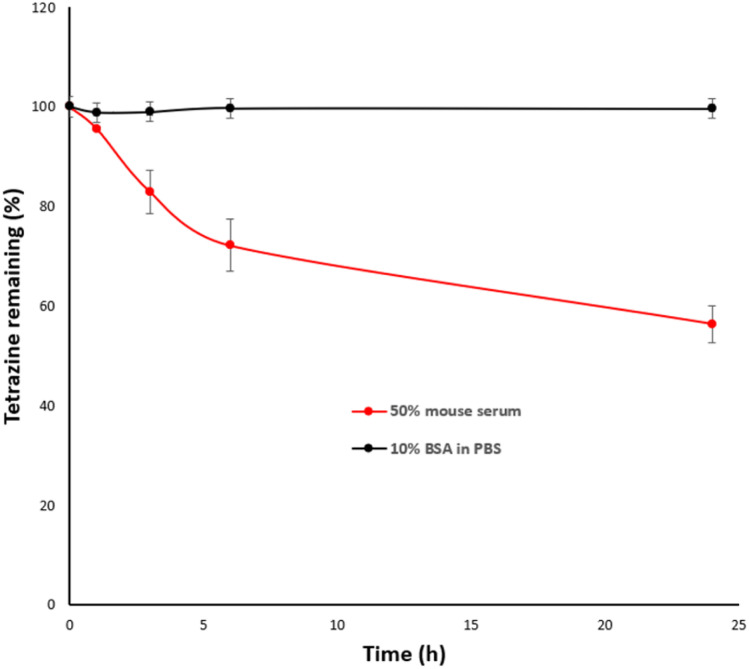
Stability study of PEG-Tz 3 in 10% BSA in PBS and in 50% mouse serum/PBS at 37 °C monitored over 24 hours at *λ* = 520 nm. Data represented as mean ± SEM (*n* = 3).

Tetrazines are reported to have limited stability *in vivo*,^15,50,51^ hence it was important to also determine the stability of the PEG-Tz activator 3. PEG-Tz 3 was therefore incubated with 10% BSA in PBS and in mouse serum at 37 °C and the amount of the remaining intact Tz was measured by HPLC over 24 hours.^10,39,52^ PEG-Tz 3 showed excellent stability in 10% BSA in PBS (99 ± 0.4% remaining intact after 24 hours incubation) and good stability in mouse serum (56 ± 3.6% remaining intact after 24 hours incubation) (Fig. 6). Overall, this suggests that conjugation of Tz to the 10 kDa PEG polymer improved its serum stability in comparison with other reported Tz activators that completely lose their stability in serum over the same period of time.^43,52^

## 3. Conclusion

A bioorthogonal prodrug activation strategy has been developed for breast cancer using PEG-based activators. Two triphenylphosphine-prodrugs and two TCO-prodrugs containing the *N*-mustard and doxorubicin cytotoxic moieties were also developed. HPLC-monitored release studies demonstrated that activation of the prodrug was dependent on the molecular weight of the PEG-activator and was slower for the Staudinger ligation compared with the Tz ligation (24 hours *versus* 20 minutes for complete release). The feasibility of using PEG-activators for prodrug activation was further validated *in vitro* in MCF-7 and MDA-MB-231 breast cancer cell lines. Very good levels of drug activity were restored upon incubation of PEG-azide with the triphenylphosphine-prodrugs (∼68–76% restoration of cytotoxicity) and full restoration of activity was achieved upon the incubation of PEG-Tz with the TCO-prodrugs (100% restoration of cytotoxicity). Moreover, conjugation of Tz to the 10 kDa PEG polymer improved its serum stability in comparison with other reported Tz activators that completely lose their stability in serum over the same period of time. These results suggest the feasibility of using PEG-based activators for bioorthogonal prodrug activation in breast cancer. Their size (10 kDa) renders them suitable for further *in vivo* application for selective cancer targeting through accumulation in solid tumours *via* the EPR effect and passive targeting.

## 4. Experimental section

### 4.1. Materials

All chemicals and solvents were purchased from Sigma Aldrich, UK unless otherwise specified. MeO-PEG-NHS (MW 11153 Da) was purchased from Iris Biotech, Germany. (4-(6-Methyl-1,2,4,5-tetrazin-3-yl)phenyl)methanamine·HCl was purchased from BLD Pharm, Germany. Compound 6 was purified by flash column chromatography using Silica gel 60 (particle size 40–63 μm). ^1^H NMR, ^13^C NMR and ^31^P NMR spectra were recorded in deuterated chloroform (CDCl_3_) using a Bruker DPX 400 (400 MHz) spectrometer. Human breast adenocarcinoma epithelial cell line (MCF-7), the triple negative breast cancer cell line (MDA-MB-231), and mouse fibroblasts (L929) were purchased from the American Type Culture Collection (ATCC, Rockville, MD, USA). FBS and trypsin were purchased from Gibco, UK.

### 4.2. Chemical synthesis

Compounds 8, 9, 10, 11 and 13 were prepared and characterized according to previously reported procedures.^11^ TCO-*N*-mustard prodrug 15 and TCO-doxorubicin prodrug 16 were prepared according to previously reported procedures.^43^

#### 4.2.1 MeO-PEG-tetrazine (3)

(4-(6-Methyl-1,2,4,5-tetrazin-3-yl)phenyl)methanamine hydrochloride (20 mg, 0.08 mmol, 4 eq.) was dissolved in anhydrous chloroform (4 mL). To the solution, *N*,*N*-diisopropyl-ethylamine (DIPEA) (29 μL, 168.29 μmol, 10 eq.) was added. MeO-PEG-NHS (MW 11153, 200 mg, 17.93 μmol, 1 eq.) was then added in portions. The reaction was stirred for 3 days, under an inert atmosphere and protected from light. The reaction mixture was condensed by evaporation under vacuum before it was poured dropwise on ice-cooled diethyl ether (30 mL). The precipitate was left on ice for 1 h and then it was filtered to obtain a pink product. The product was left to dry under vacuum. Then, the pink product was dissolved in deionised H_2_O (∼15 mL) and dialysed against deionised H_2_O (5 L) using regenerated cellulose membrane (MWCO 3500 Da). The H_2_O was changed 3 times over 24 h. The solution was then collected and freeze-dried to yield the MeO-PEG-tetrazine 3 as a pink powder (150 mg, 74%). The amount of free tetrazine (<0.2%) and the content of tetrazine (1.78% wt/wt) were determined using RP-HPLC and ^1^H NMR, respectively. ^1^H NMR (CDCl_3_, 400 MHz) *δ* 2.57–2.74 (4H, m, CO–C**H**_**2**_–C**H**_**2**_–CO), 3.09 (3H, s, C**H**_**3**_), 3.38 (3H, s, OC**H**_**3**_), 3.42–3.72 (441H, m, PEG unit), 3.78–3.86 (4H, m, C**H**_**2**_–C**H**_**2**_–OCH_3_), 4.54 (2H, d, *J* = 6.0 Hz, C**H**_**2**_-benzyl), 7.50 (2H, d, *J* = 8.5 Hz, Ar–H), 8.54 (2H, d, *J* = 8.4 Hz, Ar–H). ^13^C NMR (CDCl_3_, 100 MHz) *δ* 21.14 (CH_3_), 31.59 (CO–CH_2_–CH_2_–CO), 31.84 (CO–CH_2_–CH_2_–CO), 39.34 (PEG unit), 43.11 (CH_2_-benzyl), 59.02 (OCH_3_), 69.69 (CH_2_–CH_2_–OCH_3_), 70.54 (PEG unit), 71.91 (CH_2_–OCH_3_), 128.12 (Ar–CH), 128.31 (Ar–CH), 130.65 (Ar–C), 143.68 (Ar–C), 167.19 (Ar–C), 172.36 (C=O), 172.53 (C=O).

#### 4.2.2 4-Nitrophenyl 2-(diphenylphosphanyl) benzoate (6)^9^

2-Diphenylphosphanyl benzoic acid 5 (0.37 g, 1.20 mmol, 1eq.) was dissolved in anhydrous DCM (50 mL) under an inert atmosphere. DCC (0.25 g, 1.20 mmol, 1 eq.) and DMAP (0.07 g, 0.060 mmol, 0.5 eq.) were added to the reaction mixture which was kept under an inert atmosphere. The reaction mixture was stirred at room temperature for 30 min and then 4-nitrophenol (0.15 g, 1.20 mmol, 1 eq.) was added. The mixture was stirred overnight at room temperature. The solvent was removed under vacuum and ice-cold acetone was added to the residue to precipitate the urea by-product to be removed by filtration. After removal of acetone under vacuum, the crude product was purified using flash column chromatography (hexane/ethyl acetate, 9 : 1 v/v) to yield the 4-nitrophenyl 2-(diphenylphosphanyl) benzoate 6 as a pale yellow solid (0.16 g, 38%). m.p. 125–126 °C. ^1^H NMR (CDCl_3_, 400 MHz) *δ* 6.98–7.05 (1H, m, Ar–H), 7.06–7.13 (2H, m, 4-nitrophenyl ring Ar–H), 7.24–7.40 (10H, m, diphenyl rings Ar–H), 7.44–7. 54 (2H, m, Ar–H), 8.17–8.22 (2H, m, 4-nitrophenyl ring Ar–H), 8.22–8.28 (1H, m, Ar–H). ^13^C NMR (CDCl_3_, 100 MHz) *δ* 122.49 (Ar–CH), 125.09 (Ar–CH), 128.44 (Ar–CH), 128.65 (Ar–CH), 128.72 (Ar–CH), 129.00 (Ar–CH), 131.51 (Ar–CH), 133.07 (Ar–CH), 133.95 (Ar–CH), 134.16 (Ar–CH), 134.59 (Ar–CP), 137.15 (Ar–CP), 137.32 (Ar–C–NO_2_), 145.32 (Ar C–O), 155.28 (C=O). ^31^P NMR (CDCl_3_, 162 MHz) *δ* −3.63. IR *ν*_max_ [cm^−1^] (powder) 3042 (C=C–H), 1737 (C=O). *m*/*z* (FTMS+ESI) M^1+^ (C_25_H_19_NO_4_P) requires 428.1046. Found 428.1043. HPLC analysis: CH_3_CN (1% CH_3_COOH) – H_2_O (70 : 30 v/v), 99.05% purity.

### 4.3. HPLC release studies

Release reaction studies were measured by RP-HPLC, Hewlett-Packed Series 1100 system, with an ACE C18 reverse phase column (250 × 4.6 mm, 5 μm particle size, 300 Å pore size). UV-Vis spectrophotometers (Cary 300 Bio UV-Visible spectrophotometer and Jenway-7315 spectrophotometer) were used to record ultraviolet absorbance of the target compounds.

4-Nitrophenyl 2-(diphenylphosphanyl)benzoate 6 (0.043 mg mL^−1^) (0.1 mM) in 3 mL of CH_3_CN/H_2_O (1 : 1 v/v) was reacted with PEG-azide 10 kDa or 20 kDa or benzyl azide (0.2 mM) at 37 °C and 4-nitrophenyl carbonate TCO 14 (0.03 mg mL^−1^) (0.1 mM) in 3 mL of CH_3_CN/H_2_O (1 : 1 v/v) was reacted with PEG-Tz 3 (0.2 mM) at 37 °C. At different time intervals, samples of 25 μL were withdrawn and analysed by HPLC. The flow rate was 1 mL min^−1^, the mobile phase was 70% CH_3_CN (1% CH_3_COOH in CH_3_CN) and 30% H_2_O (1% CH_3_COOH in H_2_O) over 20 min in case of 6 with azide activators and the mobile phase was 20% of CH_3_CN increasing to 70% over 25 min, returning to 20% for 5 min in the case of 14 with 3, UV detector at *λ* = 310 nm and 254 nm together. The release profile curve for 4-nitrophenol was produced by converting AUC to concentration using the calibration curve equation. The calibration curve was obtained through preparation of a stock solution of 4-nitrophenol (1 mg mL^−1^) in CH_3_CN : H_2_O (1 : 1 v/v) and was used to prepare a range of concentrations 0.01–1 mg mL^−1^.

Triphenylphosphine-Dox prodrug 13 (0. 08 mg mL^−1^) (0.1 mM) in 3 mL of CH_3_CN/H_2_O (1 : 1 v/v) was reacted with PEG-azide 10 kDa 4 (0.2 mM) at 37 °C and TCO-Dox prodrug 16 (0.07 mg mL^−1^) (0.1 mM) in 3 mL of CH_3_CN/H_2_O (1 : 1 v/v) was reacted with PEG-Tz 3 (0.2 mM) at 37C°. At different time intervals, samples of 25 μL were withdrawn and analysed by HPLC by a gradient elution method using an aqueous gradient in CH_3_CN. The flow rate was 1 mL min^−1^, the mobile phase was 20% of CH_3_CN increasing to 70% over 25 min, returning to 20% for 5 min (UV detector at *λ* = 233 nm). The release profile curve for doxorubicin was produced by converting AUC to concentration using the calibration curve equation. The calibration curve was obtained through preparation of a stock solution of doxorubicin (1 mg mL^−1^) in CH_3_CN : H_2_O (1 : 1 v/v) and was used to prepare a range of concentrations 0.01–0.5 mg mL^−1^.

### 4.4. *In vitro* prodrug activation

MCF-7 cells were cultured in RPMI-1640 medium supplemented with 5% FBS. MDA-MB-231 and mouse fibroblasts were cultured in Dulbecco's modified eagle's medium (1 g L^−1^ glucose and 5 g L^−1^, respectively with l-glutamine) supplemented with 10% FBS.

The *in vitro* prodrug activation and antiproliferative activity of compounds 11, 13, 15 and 16 were determined using the MTT assay. MCF-7 cells or MDA-MB-231 cells were seeded on 96-well plates (4 × 10^4^ cells per mL) and (2 × 10^4^ cells per mL), respectively and incubated at 37 °C for 24 h. Cells were then treated with a range of concentration of the prodrugs 11, 13, 15 and 16 (0.001–10 μM) and respective two equivalents range of concentration of the PEG activators 3 and 4 (0.002–20 μM) and incubated for 67 h. After incubation, 20 μL of MTT in PBS solution (0.5 mg mL^−1^) were added in each well and further incubated for 5 h. The resulting formazan crystals were then dissolved in DMSO (100 μL) and incubated for 30 minutes after carefully removing the treatments-containing media and MTT solution. The absorbance was recorded at 570 nm by microplate reader (infiniteF50 TECAN). The cells without treatment were used as the control. Assays were performed in three replicates, which the statistical mean and standard error of mean were used to estimate the cell viability. IC_50_ (inhibitory concentration to induce 50% cell death) values were determined using GraphPad Prism 8.0.2 according to the fitted data.

### 4.5. Fluorescence spectra of drug–DNA complexes

A solution of TCO-Dox prodrug 16 was mixed with a solution of PEG-Tz 3 and used instantaneously. A solution of triphenylphosphine-Dox prodrug 13 was mixed with a solution of PEG-azide 4 and incubated for 24 hours at 37 °C. The molar ratio of prodrugs to PEG-activator was 1 : 2 and the final concentration of the prodrugs was 10 μM. Subsequently, the mixture was added to an equal volume of DNA (deoxyribonucleic acid sodium salt from calf thymus) aqueous solution (1 mg mL^−1^), and then the fluorescence spectrum of Dox was recorded (excitation wavelength was 488 nm and emission wavelength was 550–650 nm). Similarly, the Dox 12 or TCO-Dox 16 or triphenylphosphine-Dox 13 was added into DNA aqueous solution, and the fluorescence spectra were recorded.

### 4.6. Stability of PEG-Tz

The stability of PEG-Tz 3 was evaluated in 10% BSA in PBS and in 50% mouse serum/PBS over time. Stock solutions of 3 were prepared in PBS at a concentration of 5 mM of Tz according to the loading%, the stock solution was diluted to 0.5 mM in 50% mouse serum/PBS or 10% BSA in PBS. After incubation for various time points (0, 1, 3, 6 and 24 h), the samples were mixed with 250 μL of cold acetonitrile for extraction. The samples were then centrifuged at 1560*g* for 5 minutes and the clear supernatant was analyzed by HPLC at *λ* = 520 nm (*n* = 3).

### 4.7. Statistical analysis

Data were presented as mean ± standard error of mean. Statistical analysis was carried out for active Dox 12 against Dox prodrugs 13 and 16 and Dox prodrugs 13 and 16 after addition of PEG-azide 4 and PEG-Tz 3, respectively on MCF-7 cells and MDA-MB-231 cells, and for *N*-mustard prodrugs 11 and 15 against *N*-mustard prodrugs 11 and 15 after addition of PEG-azide 4 and PEG-Tz 3, respectively on MCF-7 cells and MDA-MB-231 cells by one-way ANOVA followed by Bonferroni *post hoc* test using GraphPad Prism 8.0.2 software and statistical significance was set at *p* < 0.05 (specifically, * for *p* < 0.05; ** for *p* < 0.01; *** for *p* < 0.001).

## Data availability

The data supporting this article have been included as part of the ESI.†

## Conflicts of interest

There are no conflicts to declare.

## Supplementary Material

RA-015-D4RA08758E-s001
