# Supplementary material for: Polymeric PEG-based bioorthogonal triggers for prodrug activation in breast cancer
Source: RSC Adv. 2025 Mar 5;15(9):7127–38. doi: 10.1039/d4ra08758e (PMC11881796; doi:10.1039/d4ra08758e)
Supplement: RA-015-D4RA08758E-s001 [file RA-015-D4RA08758E-s001.pdf]

Supplementary Information (SI) for RSC Advances.
This journal is © The Royal Society of Chemistry 2025

Supporting Information

Polymeric PEG-based bioorthogonal triggers for prodrug activation in breast cancer

Madonna M. A. Mitry,[a,b] Helen M.I. Osborn,[a*] Francesca Greco[a*]

[a] Reading School of Pharmacy, University of Reading, Whiteknights, Reading, RG6 6AD. UK

[b] Dept. of Pharmaceutical chemistry, Faculty of Pharmacy, Ain Shams University, Cairo, 11566. Egypt

*Corresponding author, E-mail: h.m.i.osborn@reading.ac.uk , f.greco@reading.ac.uk

Table of contents

IC_{50} of Dox prodrugs 13 and 16 and *N*-mustard prodrugs 11 and 15 against MCF-7 cells and MDA-MB-231 cells………… 2

IC_{50} of Dox prodrugs 13 and 16 and *N*-mustard prodrugs 11 and 15 against L929 cells… 3

^{1}H, ^{13}C, and ^{31}P NMR spectra and mass spectra………… 4

HPLC chromatographs of 4-nitrophenol and Dox release profiles………… 8

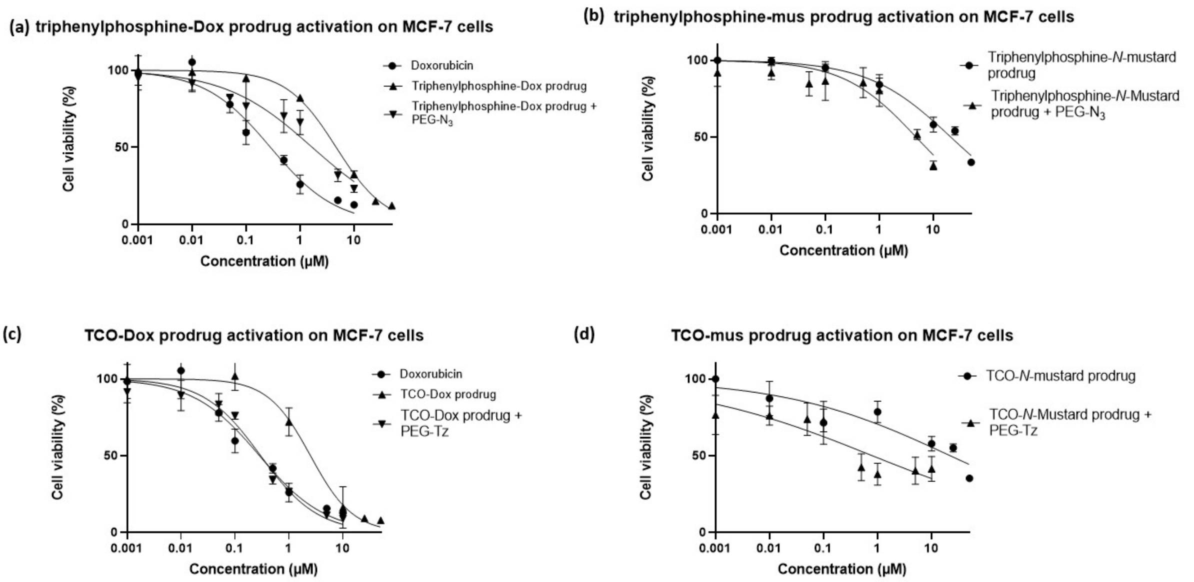

Figure *S*1: a) Cytotoxicity profile of Dox **12**, triphenylphosphine-Dox prodrug **13** and prodrug **13** after activation by PEG-azide **4** against MCF-7 cells. b) Cytotoxicity profile of triphenylphosphine-*N*-mustard prodrug **11** and prodrug **11** after activation by PEG-azide **4** against MCF-7 cells. c) Cytotoxicity profile of Dox **12,** TCO-Dox prodrug **16** and prodrug **16** after activation by PEG-Tz **3** against MCF-7 cells. d) Cytotoxicity profile of TCO-*N*-mustard prodrug **15** and prodrug **15** after activation by PEG-Tz **3** against MCF-7 cells. Data are presented as mean ± SEM (n=3).

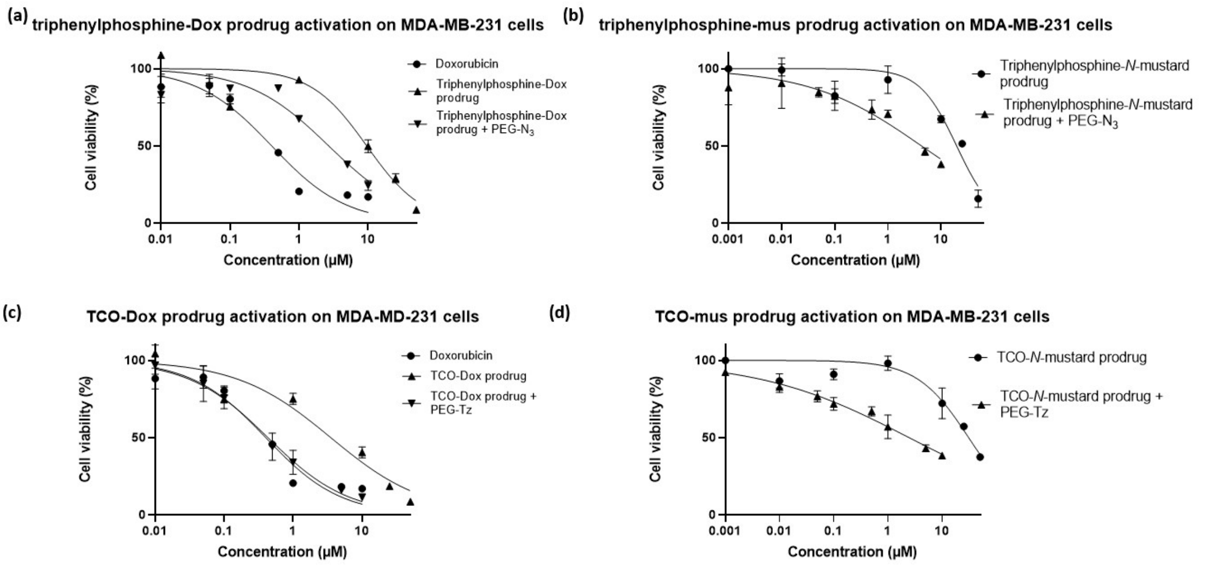

Figure *S*2: a) Cytotoxicity profile of Dox **12**, triphenylphosphine-Dox prodrug **13** and prodrug **13** after activation by PEG-azide **4** against MDA-MB-231 cells. b) Cytotoxicity profile of triphenylphosphine-*N*-mustard prodrug **11** and prodrug **11** after activation by PEG-azide **4** against MDA-MB-231 cells. c) Cytotoxicity profile of Dox **12,** TCO-Dox prodrug **16** and prodrug **16** after activation by PEG-Tz **3** against MDA-MB-231 cells. d) Cytotoxicity profile of TCO-*N*-mustard prodrug **15** and prodrug **15** after activation by PEG-Tz **3** against MDA-MB-231 cells. Data are presented as mean ± SEM (n=3).

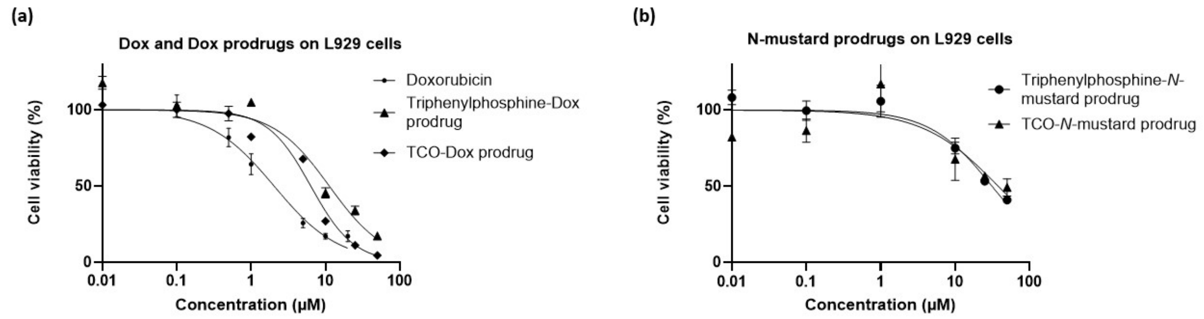

Figure *S*3: a) Cytotoxicity profile of Dox **12**, triphenylphosphine-Dox prodrug **13** and TCO-Dox prodrug **16** against L929 cells. b) Cytotoxicity profile of triphenylphosphine-*N*-mustard prodrug **11** and TCO-*N*-mustard prodrug **15** against L929 cells. Data are presented as mean ± SEM (n=3).

^{1}H and ^{13}C NMR Spectra

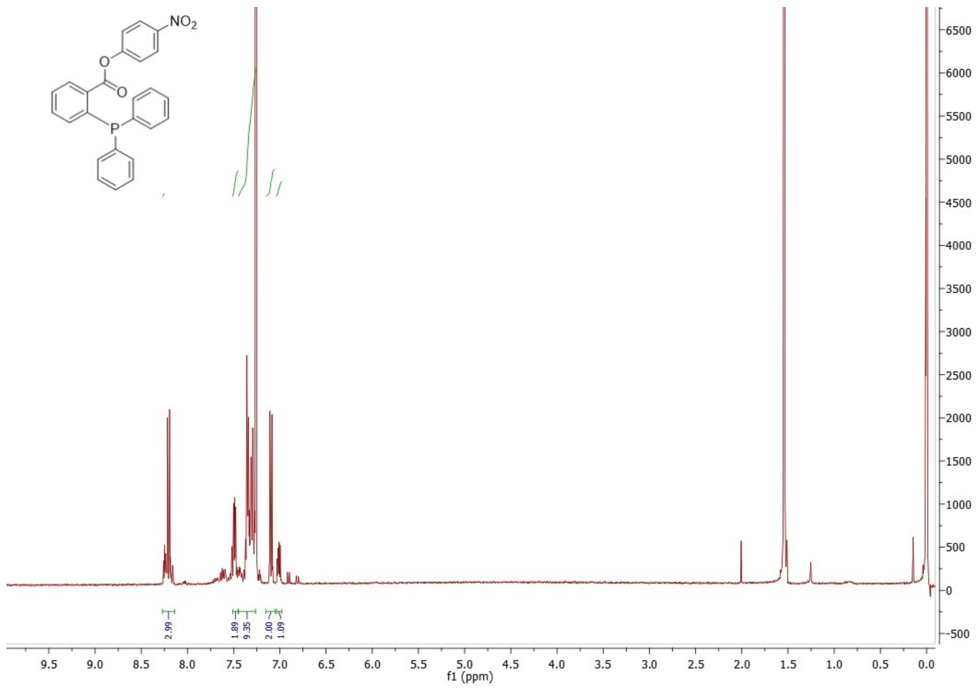

^{1}H NMR spectrum (400 MHz, $CDCl_3$) of compound **6**

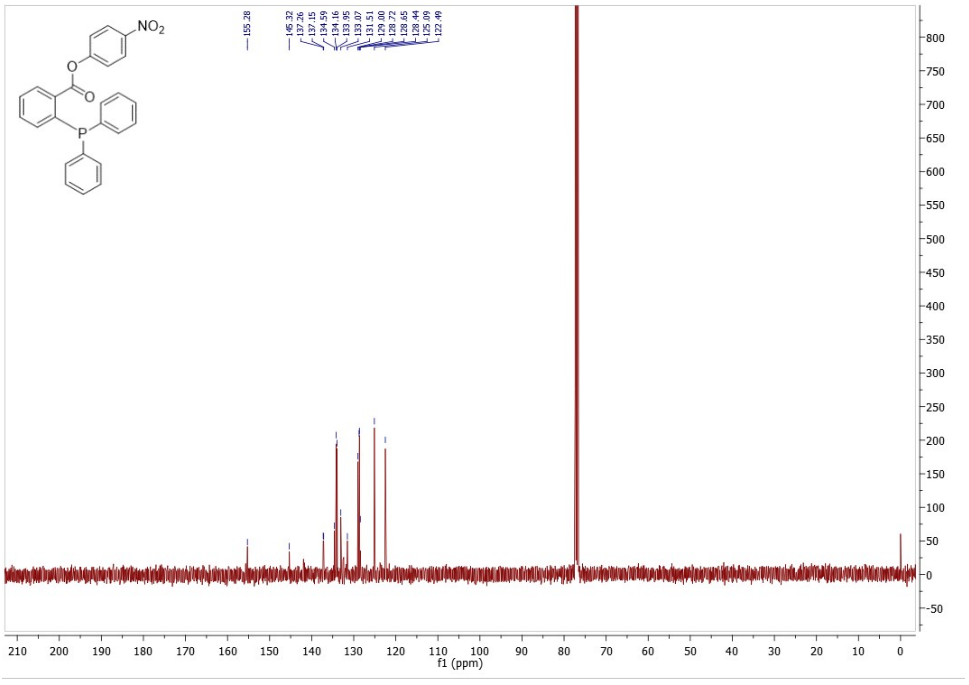

^{13}C NMR spectrum (100 MHz, $CDCl_3$) of compound **6**

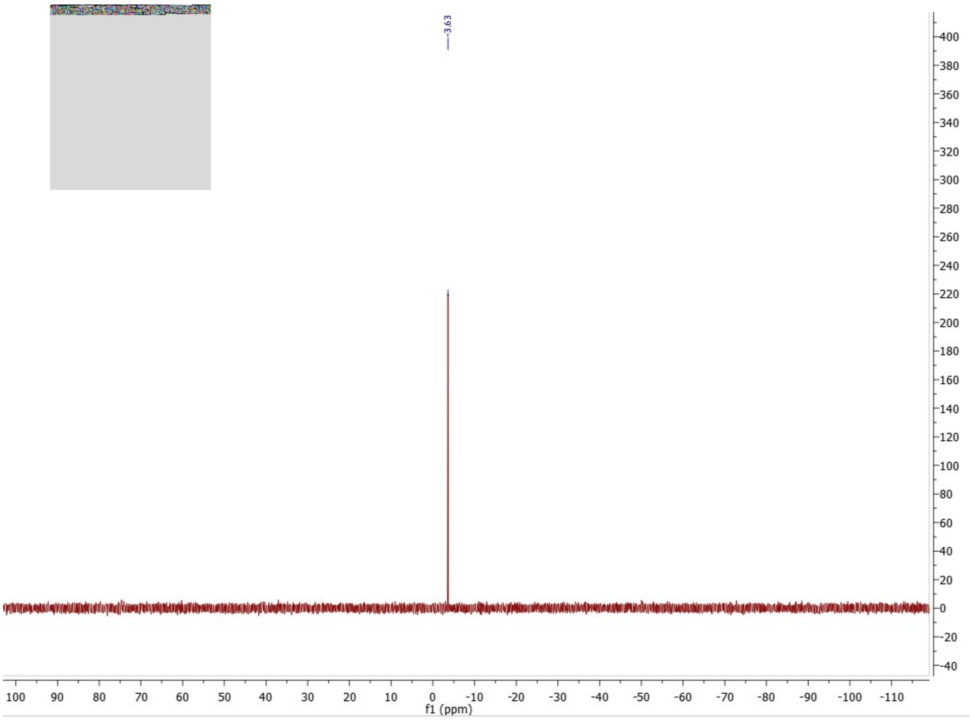

^{31}P NMR spectrum (162 MHz, $CDCl_3$) of compound **6**

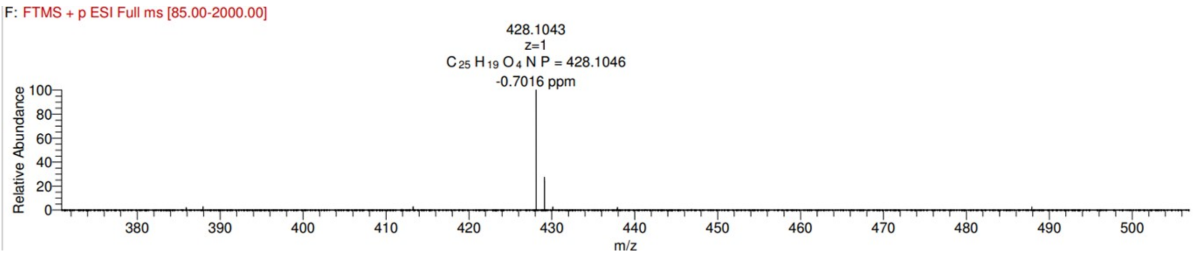

Mass spectrum of compound **6**

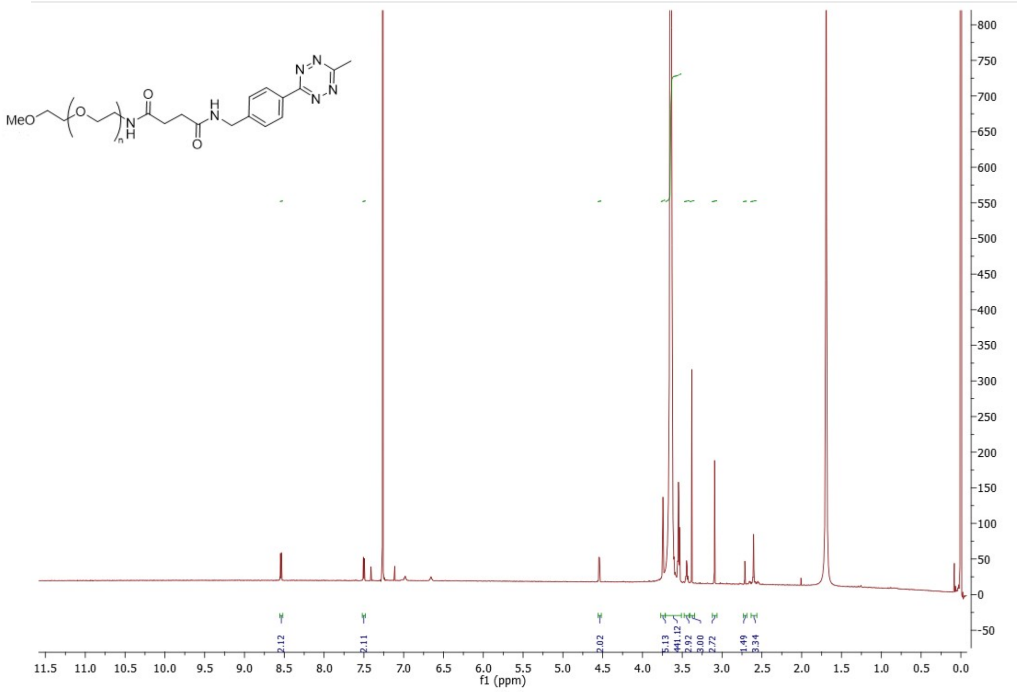

^1H NMR spectrum (400 MHz, $CDCl_3$) of compound **3**

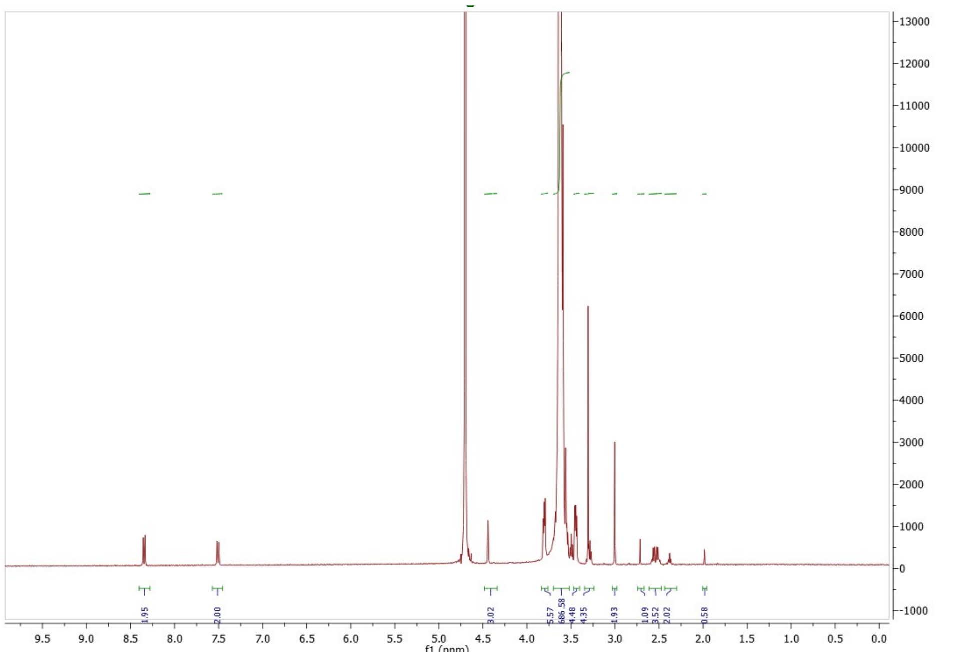

^1H NMR spectrum (400 MHz, D_2O) of compound **3**

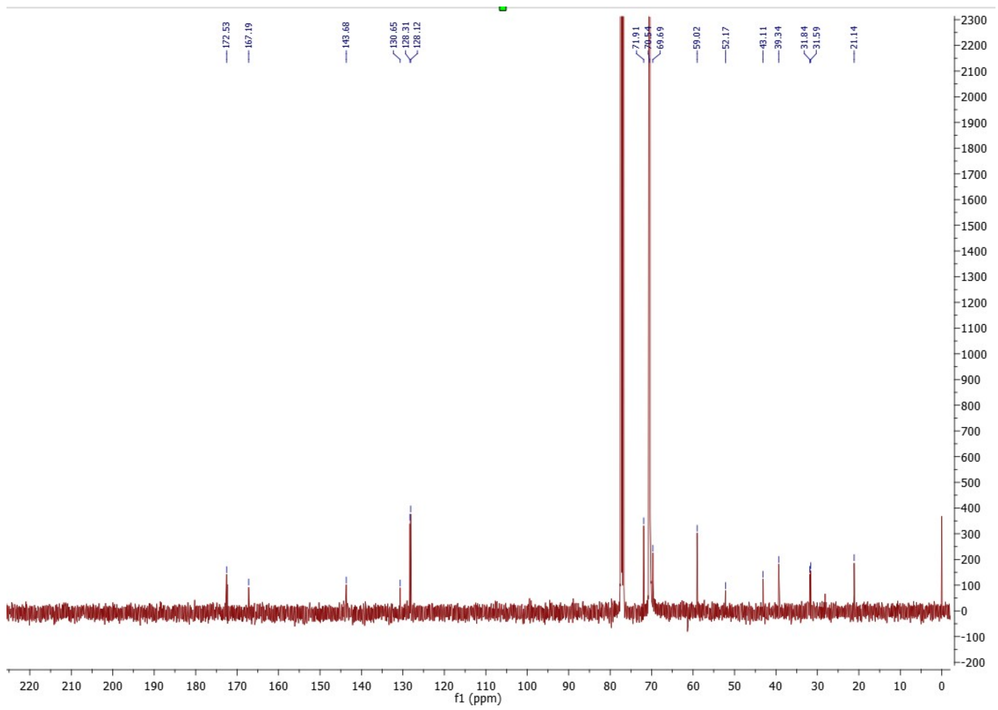

^{13}C NMR spectrum (100 MHz, $CDCl_3$) of compound **3**

Data for Compounds **11, 13, 15, 16** have been previously reported by ourselves within M. M. A. Mitry, S. Y. Boateng, F. Greco and H. M. I. Osborn, *RSC Med. Chem*., 2023, **14**, 1537–1548; M. M. A. Mitry, M. L. Dallas, S. Y. Boateng, F. Greco and H.M.I. Osborn, *Bioorg. Chem*., 2024, **147**, 107304.

HPLC chromatograms of release profile of 4-nitrophenol and Doxorubicin

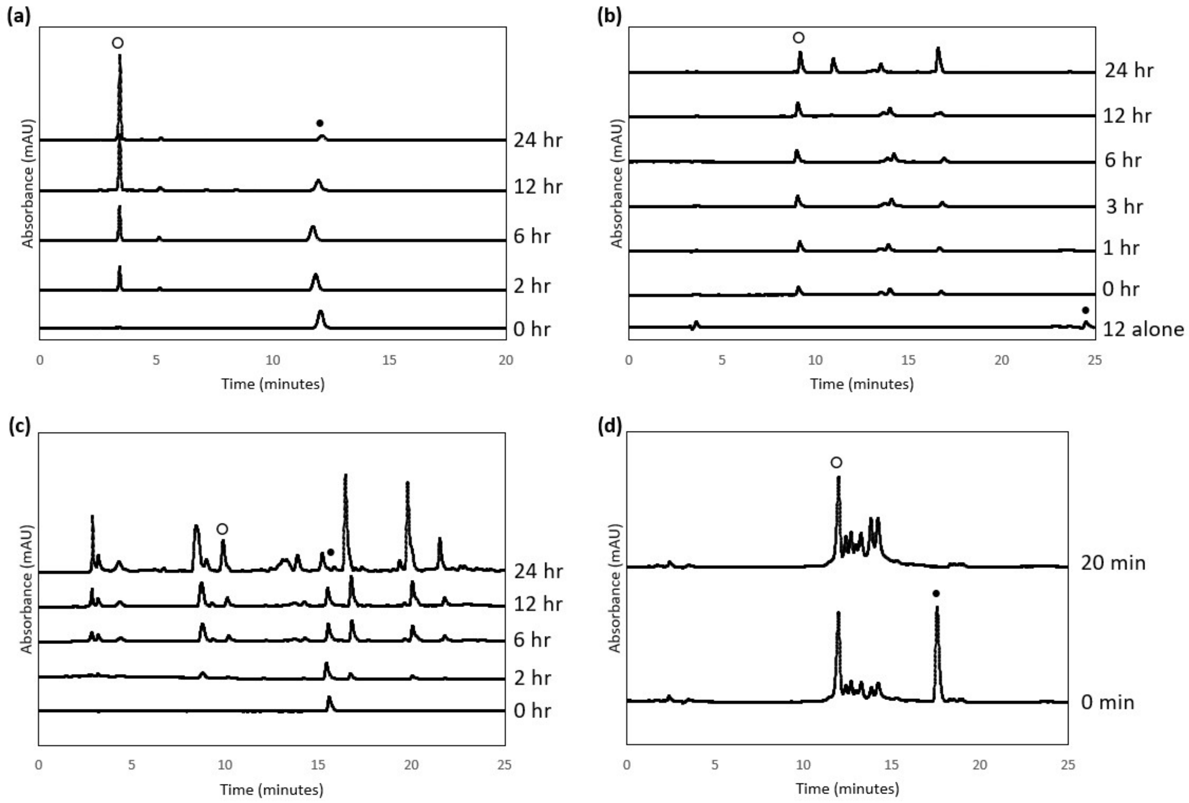

a) HPLC chromatograph of 4-nitrophenol release from triphenylphosphine ester model prodrug **6** by PEG-azide (10 KDa) **4** at 37 °C in aqueous MeCN (1:1) as a function of time. Legend: •: triphenylphosphine ester model prodrug **6**; ○: 4-nitrophenol.

b) HPLC chromatograph of 4-nitrophenol release from TCO carbonate model prodrug **14** by PEG-Tz (10 KDa) **3** at 37 °C in aqueous MeCN (1:1) as a function of time. Legend: •: TCO carbonate model prodrug **14**; ○: 4-nitrophenol.

c) HPLC chromatograph of doxorubicin **12** release from triphenylphosphine-Dox prodrug **13** by PEG-azide (10 KDa) **4** at 37 °C in aqueous MeCN (1:1) as a function of time. Legend: •: triphenylphosphine-Dox prodrug **13**; ○: doxorubicin **12**.

d) HPLC chromatograph of doxorubicin **12** release from TCO-Dox prodrug **16** by PEG-Tz **3** at 37 °C in aqueous MeCN (1:1) as a function of time. Legend: •: TCO-Dox prodrug **16**; ○: doxorubicin **12**
